# The Effect of Mineral Trioxide Aggregate Mixed with Chlorhexidine as Direct Pulp Capping Agent in Dogs Teeth: A Histologic Study

**DOI:** 10.22037/iej.2016.12

**Published:** 2016

**Authors:** Hamed Manochehrifar, Masoud Parirokh, Sina Kakooei, Mohammad Mehdi Oloomi, Saeed Asgary, Mohammad Jafar Eghbal, Fatemeh Mashhadi Abbas

**Affiliations:** a *Endodontic Department, Dental School, Kerman University of Medical Sciences, Kerman, Iran; *; b * Oral and Dental Diseases Research Center, Dental School, Kerman University of Medical Sciences, Kerman, Iran; *; c * Department of Clinical Sciences, Veterinary Medicine School, Shahid Bahonar University of Kerman, Iran; *; d *Iranian Center for Endodontic Research, Research Institute of Dental Sciences, Dental School, Shahid Beheshti University of Medical Sciences, Tehran, Iran; *; e * Dental Research Center, Research Institute of Dental Sciences, Dental School, Shahid Beheshti University of Medical Sciences, Tehran, Iran;*; f *Endodontic Department, Shahid Beheshti Dental School, Tehran, Iran*

**Keywords:** Chlorhexidine, Mineral Trioxide Aggregate, Pulp Capping, Vital Pulp Therapy

## Abstract

**Introduction:**

The aim of the present investigation was to compare the efficacy of mineral trioxide aggregate (MTA) and 0.2% chlorhexidine (CHX) mixture to pure MTA, as a pulp capping material.

**Methods and Materials:**

The pulp of 24 lateral incisors and canines from four dogs were exposed and capped either with MTA or MTA+0.2% CHX. After 2 months the animals were sacrificed and the teeth were prepared for histological evaluation in terms of calcified bridge formation, the degree of inflammation and presence of necrosis. The Fisher’s exact test was used for data analysis.

**Results:**

The results showed that formation of complete calcified bridge in MTA specimens was significantly more than MTA+CHX (*P*<0.05). No significant difference was found in the degree of inflammation and necrosis between MTA and MTA+CHX groups (*P*>0.05).

**Conclusion:**

Mixing MTA with CHX as pulp capping agent had a significant negative impact on formation of calcified bridge on directly capped dog’s teeth.

## Introduction

Mineral trioxide aggregate (MTA) is the material of choice for several clinical applications such as vital pulp therapy (pulp capping, partial and total pulpotomy), root-end filling, perforation repair and apical barrier formation for immature permanent teeth with necrotic pulps [1]. Numerous investigations have been performed on various aspects of MTA from physical and chemical properties to antibacterial activity, biocompatibility, sealing ability, and clinical applications. The results of these investigations have shown that MTA is a bioactive material which is biocompatible and has excellent sealing ability [1-3]. 

Despite all the advantages of MTA in terms of biocompatibility and sealing ability, the material has several drawbacks such as long setting time, discoloration potential, difficult handling characteristics and high cost [4]. In addition, several investigations have shown that MTA has limited antibacterial properties [5-8]. For that reason it seems reasonable to mix antibacterial agents with MTA to improve its antibacterial properties [6, 9]. 

Several investigations have tried to improve various properties of MTA by mixing the material with chlorhexidine (CHX), various concentration of NaOCl, K-Y jelly and various concentration of CaCl_2_ [9-22]. Recent investigations confirmed that selected physical properties of MTA have been improved when mixed with CHX [23, 24]. The results of two separate investigations that mixed MTA with two different concentrations of CHX have confirmed that the antibacterial effect of the material significantly improved in comparison with the mixture of MTA with sterile water [11, 15].

Several animal and human investigations on vital pulp therapy have shown that MTA can be successfully used as pulp capping material [25-34]. However, none of the previous investigations that used MTA as pulp capping agent used the material mixed with CHX. Previous investigations have only evaluated physical properties, sealing abilities or biocompatibility of MTA mixed with CHX; therefore, the aim of the present investigation was to compare the efficacy of MTA+CHX mixture as direct pulp capping agent on dogs’ teeth in comparison with plain MTA.

## Materials and Methods

The protocol of the study was approved by the Ethics Committee of Kerman University of Medical Sciences (Grant No.: K/A-90-280). In the present study, 24 sound lateral incisors and canines from 4 dogs were used. After initial sedation with ketamine HCl (Alfasan, Woerden, The Netherlands) and xylazine (Alfasan International, Woerden, The Netherlands) animals were intubated and inhalation sedation with halothane was used for anesthesia throughout the dental procedure. Local anesthesia was achieved with 3% prilocaine (Prilonest; DFL Industria, Rio de Janeiro, Brazil). Subsequently, both maxillary and mandibular lateral incisors and canine teeth were washed with 0.2% CHX. In each tooth a standard class V cavity was prepared with a new sterile diamond bur (Tizkavan, Tehran, Iran) with copious water spray at the cervical part and 0.5 to 1-mm exposure zones were produced in each tooth. Immediately following the exposure the cavity was washed with sterile 0.9% normal saline and the exposed pulp was capped with either grey ProRoot MTA (Dentsply, Tulsa Dental, Tulsa, OK, USA) mixed with sterile water or MTA mixed with 0.2% CHX (Shahre Daru, Tehran, Iran). The coronal cavity was immediately restored with glass-ionomer cement (GC Corp., Kyoto, Japan).

After two months the animals were sacrificed with overdose of the anesthetic solution and after vital perfusion with 10% formalin the teeth and the surrounding tissues were removed as block sections. All the specimens were kept in 10% formalin for 2 weeks and then decalcified with 10% formic acid for 4 months. Then the specimens were prepared for Hematoxylin and Eosin staining. Evaluation of the specimens was based on the following criteria used in a previous investigation [33]: calcified bridge formation (0=incomplete and 1=complete); presence of inflammatory cells beneath the capping area [0=absent to mild (scattered chronic inflammatory cells) and 1=moderate to severe (obvious number of chronic/acute inflammatory cells)], and pulp necrosis beneath the capping area [0=absent and 1=present]. Data was analyzed with Fischer’s exact test. The level of significance was set at 0.05.

## Results

Six teeth were excluded; two due to defective restoration and four because of technical problems during tissue preparation for histological evaluation. Therefore, finally 18 teeth (10 in MTA and 8 in MTA+CHX groups) were evaluated. 

**Figure 1:**
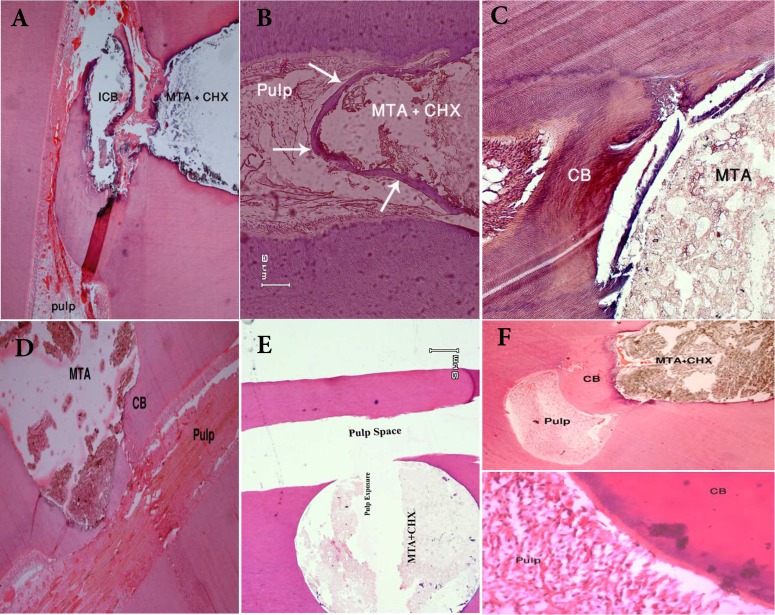
*A)* Incomplete calcified bridge (ICB) beneath the capping area in a tooth treated with MTA+CHX, *B)* Calcified bridge (white arrows) in a tooth capped with MTA+CHX, *C)* Complete calcified bridge (CB) formation in a tooth capped with MTA, *D)* Formation of calcified bridge beneath MTA as pulp capping agent, *E)* Formation of total necrosis beneath MTA+CHX as pulp capping agent, *F)* Presence of inflammatory cell in pulp cap with MTA+CHX despite formation of calcified bridge

The calcified bridge formation in the MTA group was significantly higher in MTA samples compared to the MTA+CHX specimens (*P*<0.05). Both MTA and MTA+CHX specimens showed necrosis and inflammation in some of the specimens (Table 1) (Figures 1); however the differences between the groups were not significant (*P*>0.05). Most of the observed necrosis was local and were beneath the calcified bridge. All the specimens were free of polymorphonuclear infiltration that represented acute inflammation. In specimens with inflammatory cells, mostly lymphocytes and macrophages were detected. 

## Discussion

The results of the present *in vivo* study showed that the formation of a complete calcified bridge was negatively influenced when MTA+CHX was used as pulp capping agent.

In the present study, 0.2% CHX was used as a liquid to mix with MTA powder because a previous investigation on physical properties of the material reported that when 2% of CHX was mixed with MTA most of the specimens did not set even 7 days following mixing [9]. The reason for mixing CHX with MTA was to improve the material’s antibacterial activity. According to a previous laboratory investigation, mixing 0.12% CHX with MTA resulted in significantly higher antibacterial activity compared to MTA mixed with distilled water [15] and in this study gray MTA was used because a previous study showed that white MTA mixed with CHX exhibits higher inflammatory response in comparison with gray formulation [35].

**Table 1:** Comparison between MTA and MTA mixed with CHX by using histologic criteria

**MTA+CHX (** ***n*** **=8)**						**MTA (** ***n*** **=10)**					
**Inflammation (N)**		**Calcified bridge (N)**		**Necrosis (N)**		**Inflammation (N)**		**Calcified bridge (N)**		**Necrosis (N)**	
(+)	(-)	(+)	(-)	(+)	(-)	(+)	(-)	(+)	(-)	(+)	(-)
3 (37.5)	5 (62.5)	3 (37.5)	5 (62.5)	4 (50)	4 (50)	3 (30)	7 (70)	9 (90)	1 (10)	4 (40)	6 (60)

The results of the present study also showed that mixing CHX with MTA had a significant adverse effect on formation of a complete calcified bridge following pulp capping. It has been previously shown that developing new modifications of a material to improve some of its properties by adding or mixing various elements does not necessarily improve its other properties [33]. In fact, improving some properties of the material may have deleterious effects on other properties. Therefore, clinical effectiveness of the modification in a given material in addition to its physical properties, antibacterial activity, sealing ability and biocompatibility should be considered. 

Previous investigations on MTA as a pulp capping agent have shown a thicker and more uniform calcified bridge beneath MTA in comparison to other pulp capping materials such as calcium hydroxide [28, 29, 32]. Moreover, almost all specimens capped with MTA showed the formation of calcified bridge [25-33]. The present study showed that 62.5% (5 out of 8) of the specimens in the MTA+CHX group did not show complete calcified bridge, whereas only 10% of the specimens (1 out of 10) in the MTA group showed the same event. 

Biocompatibility tests on MTA mixed with CHX showed rare results. MTA mixed with 0.12% CHX resulted in an increase in cell apoptosis compared to MTA and sterile water [14], whereas the results of an experiment on the biocompatibility of MTA mixed with CHX showed that the mixture was well tolerated when implanted subcutaneously [11]. The presence of necrosis and inflammation following the use of MTA as a pulp capping material has been reported by several investigations [31, 32, 36]. In the present study, no significant differences were found between MTA+CHX and MTA specimens. Meanwhile, an *in vivo* investigation on biocompatibility of MTA+CHX reported that adding CHX to MTA did not affect the material’s biocompatibility [11].

Significantly lower number of formed calcified bridges in teeth capped with MTA+CHX can be attributed to several factors such as the difference in the calcium ion release of the material, defects in the formation of calcium hydroxide and a change in the pH of the material. MTA is a bioactive material and several investigations have confirmed that the material releases calcium and is able to stimulate releasing signaling molecules and cytokines from the tissues that may be important in calcified bridge formation [37-39]. So far, no study has been performed on the amount of calcium ion released and the capacity of MTA to release signaling molecule and cytokines when the material is mixed with CHX. There is also no study on the alteration of calcium hydroxide formation and pH changes after mixing MTA with CHX. 

Several animal investigations have reported necrosis and inflammation in the pulps capped with MTA [25-28, 33, 40, 41]. The results of the present study were in accordance with the previous investigations regarding the presence of inflammation and necrosis in the pulp space following pulp capping with MTA.

## Conclusion

In conclusion, the results of the present study showed that mixing CHX with MTA may adversely affect complete calcified bridge formation following pulp capping.
